# Association between the Zhejiang University index and hyperuricemia among adults with type 2 diabetes in China

**DOI:** 10.3389/fendo.2026.1836849

**Published:** 2026-04-23

**Authors:** Haoxiang Li, Xiaoyan Ma, Xia Deng, Yu Qin, Jiawei Wang, Ling Yang, Guoyue Yuan

**Affiliations:** 1 Department of Endocrinology and Metabolism, Institute of Endocrine and Metabolic Diseases, Affiliated Hospital of Jiangsu University, Jiangsu University, Zhenjiang, Jiangsu, China; 2 Department of Obstetrics and Gynecology, Affiliated Hospital of Jiangsu University, Jiangsu University, Zhenjiang, Jiangsu, China

**Keywords:** cross-sectional study, hyperuricemia, metabolic risk, type 2 diabetes mellitus, ZJU index

## Abstract

**Purpose:**

The Zhejiang University (ZJU) index is an established surrogate marker for metabolic dysfunction-associated steatotic liver disease (MASLD). We aimed to investigate the association between ZJU index and hyperuricemia in patients with type 2 diabetes mellitus (T2DM).

**Methods:**

We performed a retrospective cross-sectional analysis of 1772 adults with T2DM at the Affiliated Hospital of Jiangsu University. The ZJU index was derived from clinical metabolic variables, and participants were classified by hyperuricemia status. Associations between the ZJU index and hyperuricemia were examined using multivariable regression, restricted cubic spline modeling, and subgroup analyses.

**Results:**

Patients with hyperuricemia exhibited significantly worse metabolic profiles, including higher ZJU index levels (P<0.001). The ZJU index demonstrated a significant positive association with the prevalence of hyperuricemia (OR = 1.036, 95%CI: 1.017-1.056, P<0.001) and serum uric acid levels (β=1.187, 95%CI: 0.595-1.780, P<0.001), with a graded increase observed across quantiles (P for trend<0.001). Restricted cubic spline analysis further indicated a linear relationship. The association showed overall consistency across subgroups, while a stronger effect was evident in participants with coronary heart disease.

**Conclusions:**

The ZJU index is associated with hyperuricemia in T2DM and may reflect interlinked hepato-metabolic pathways that drive hyperuricemia.

## 1 Introduction

Hyperuricemia, defined as an abnormal elevation in serum uric acid (SUA) levels, has become a growing public health concern (1). Uric acid represents the product of purine metabolism, and its accumulation may occur due to excessive production or impaired excretion (2). Previous studies have demonstrated that hyperuricemia is not only a major determinant of gout but is also linked to various chronic diseases, including hypertension, diabetes, metabolic syndrome, chronic kidney disease, and cardiovascular disorders (3–5). Among patients with diabetes, hyperuricemia occurs more frequently than in the general population and has been implicated in the development of diabetic complications (6, 7). Thus, identifying determinants of uric acid levels in diabetic individuals is essential for effective prevention and management of hyperuricemia.

The Zhejiang University index (ZJU index) is a composite metabolic indicator derived from several clinical parameters, including body mass index (BMI), fasting plasma glucose (FPG), triglyceride (TG) levels, and the alanine aminotransferase to aspartate aminotransferase ratio (ALT/AST ratio) (8). Previous studies have reported that the ZJU index has been shown to be associated with metabolic dysfunction-associated steatotic liver disease (MASLD) in Chinese populations (9, 10). With the growing number of related studies, evidence suggests that the ZJU index is associated not only with MASLD but also with a variety of metabolic disorders, such as gestational diabetes mellitus, diabetic kidney diseases, hypertension, cardiovascular diseases, depression, sarcopenia, and mortality (8, 11–15). These findings imply that the ZJU index may serve as an indicator of broader metabolic dysfunction rather than being limited to liver-related conditions. Although the ZJU index has shown clinical relevance in relation to MASLD and other metabolic abnormalities, its relationship with hyperuricemia in patients with type 2 diabetes mellitus (T2DM) remains inadequately explored. Mechanistically, several components of the ZJU index-including hyperglycemia, dyslipidemia, and altered liver enzyme levels-have been linked to the development of hyperuricemia, suggesting a potential association between the two (16–18).

Therefore, this study employs a retrospective cross-sectional design involving adults with T2DM from the Affiliated Hospital of Jiangsu University, aiming to further investigate the association between the ZJU index and hyperuricemia.

## 2 Materials and methods

### 2.1 Study cohort

Data were retrospectively collected and analyzed from adult patients (≥18 years) with T2DM who were admitted to the Department of Endocrinology at the Affiliated Hospital of Jiangsu University between June 2019 and September 2022. A total of 1772 patients were included, among whom 1077 were male and 695 were female. All patients fulfilled the diagnostic criteria for T2DM recommended by the American Diabetes Association. Patients were excluded if they had: (1) other forms of diabetes; (2) acute or severe diabetic complications; (3) severe chronic infectious diseases; (4) malignancies; (5) severe hepatic or renal disease; or (6) autoimmune disorders. This study was conducted in accordance with the Declaration of Helsinki and was approved by the Ethics Committee of the Affiliated Hospital of Jiangsu University (KY2026K0209).

### 2.2 Data collection

Standardized anthropometric measurements, including height and body weight were obtained for all participants by trained physicians using calibrated equipment. BMI was derived from the formula BMI = body weight (kg)/height² (m²). Demographic information such as age, sex, educational background, and annual household income was collected through questionnaire-based interviews. Participants fasted for 8–12 hours before undergoing an oral glucose tolerance test (OGTT). FPG and 2−hour plasma glucose (2hPG) were analyzed using the glucose oxidase method. Hemoglobin A1c (HbA1c) concentrations were measured using high−performance liquid chromatography. Blood lipids [TG, total cholesterol (TC), high-density lipoprotein cholesterol (HDL), and low-density lipoprotein cholesterol (LDL)] and markers of liver [alanine aminotransferase (ALT), aspartate aminotransferase (AST), and albumin] and kidney function [serum creatinine (Scr) and SUA] were evaluated using a BEKMAN AU5800 automated biochemical analyzer. SUA was measured on the Beckman AU5800 using a uricase-based enzymatic (colorimetric) assay, with absorbance read by the instrument and results automatically calculated as uric acid concentration. The estimated glomerular filtration rate (eGFR) was derived from Scr measurements employing the Chronic Kidney Disease Epidemiology Collaboration (CKD-EPI) formula (19). Medical history-including conditions such as dyslipidemia, hypertension, and coronary heart disease-was collected based on patient self-report.

### 2.3 ZJU index calculation and hyperuricemia definition

The ZJU index was determined using the following formula: FPG (mmol/L) + BMI (kg/m²) + 3 × (ALT/AST ratio) + TG (mmol/L), with an extra score of 2 assigned to female participants. Hyperuricemia was defined by any of the following: self-reported hyperuricemia, current use of medications aimed at lowering uric acid, or elevated SUA concentrations (≥420 μmol/L in men or ≥360 μmol/L in women).

### 2.4 Statistical analysis

All statistical analyses in this research were performed using R software (version 4.5.1). For continuous variables, results were reported as mean ± standard deviation (SD) or median (interquartile range, IQR) based on distributional characteristics; categorical variables were summarized as frequencies (percentages). Comparisons between groups (hyperuricemia vs. non-hyperuricemia) were conducted using the independent samples t-test or Mann-Whitney U test for continuous variables, and the chi-square test for categorical variables. Participants were further stratified according to ZJU index quantiles. Differences among groups were assessed using one-way analysis of variance or the Kruskal-Wallis test, as appropriate. Correlations between the ZJU index and clinical parameters were evaluated using Spearman correlation analyses. Multivariable logistic regression analysis was performed to assess the association between the ZJU index and prevalence of hyperuricemia, with results presented as odds ratios (ORs) and 95% confidence intervals (CIs). Sequential models were constructed with adjustment for potential confounding factors. SHapley Additive exPlanations (SHAP) were used as a *post hoc* interpretability tool to quantify and visualize the contribution of the ZJU index and other covariates to prevalence of hyperuricemia based on the fitted multivariable logistic regression model. To further explore the potential nonlinear relationship between the ZJU index and prevalence of hyperuricemia, restricted cubic spline analysis was performed using a logistic regression framework with four knots. In addition, linear regression analysis was used to evaluate the relationship between the ZJU index and SUA levels. Receiver operating characteristic (ROC) curve analyses were performed to evaluate the discriminative ability of the ZJU index for hyperuricemia. A two-sided P<0.05 was considered statistically significant.

## 3 Results

### 3.1 Baseline characteristics

A total of 1772 participants were analyzed, including 283 (15.97%) with hyperuricemia (**Table 1**). Compared with those without hyperuricemia, affected individuals were younger, had higher BMI, and differed in education level, while sex and income were similar. They also showed higher prevalence of hypertension and dyslipidemia, with no difference in coronary heart disease. Biochemically, the hyperuricemia group had elevated ALT, AST, albumin, Scr, TG, TC, along with lower eGFR and HDL (all P<0.05). FPG and HbA1c were comparable, whereas 2hPG was slightly lower. Notably, the ZJU index was significantly higher in this group (P<0.001). Across increasing quartiles of the ZJU index, age decreased, while income, BMI, glycemic measures (FPG, 2hPG, HbA1c), liver enzymes (ALT, AST), lipid parameters (TG, TC, LDL), eGFR, SUA, and the prevalence of hypertension, dyslipidemia, and hyperuricemia increased progressively; HDL showed a decreasing trend (all P<0.05) (**Table 2**).

**Table 1:** Baseline characteristics by hyperuricemia status.

Characteristic	Overall (n=1772)	Non-Hyperuricemia (n=1489)	Hyperuricemia (n=283)	*P* value
Age (years)	54.96 ± 11.55	55.38 ± 11.16	52.72 ± 13.21	<0.001
Sex				0.232
Female	695 (39.22%)	593 (39.83%)	102 (36.04%)	
Male	1077 (60.78%)	896 (60.17%)	181 (63.96%)	
Education				0.003
Below high school	880 (49.66%)	762 (51.18%)	118 (41.70%)	
High school and above	892 (50.34%)	727 (48.82%)	165 (58.30%)	
Annual household income				0.176
≤100000	973 (54.91%)	828 (55.61%)	145 (51.24%)	
>100000	799 (45.09%)	661 (44.39%)	138 (48.76%)	
BMI (kg/m2)	24.90 ± 3.60	24.59 ± 3.41	26.56 ± 4.12	<0.001
Hypertension				<0.001
No	916 (51.69%)	812 (54.53%)	104 (36.75%)	
Yes	856 (48.31%)	677 (45.47%)	179 (63.25%)	
Dyslipidemia				<0.001
No	1136 (64.11%)	997 (66.96%)	139 (49.12%)	
Yes	636 (35.89%)	492 (33.04%)	144 (50.88%)	
Coronary heart disease				0.121
No	1642 (92.66%)	1386 (93.08%)	256 (90.46%)	
Yes	130 (7.34%)	103 (6.92%)	27 (9.54%)	
FPG (mmol/L)	10.31 ± 3.43	10.36 ± 3.44	10.03 ± 3.35	0.134
2hPG (mmol/L)	18.92 ± 5.32	19.05 ± 5.31	18.29 ± 5.36	0.029
HbA1c (%)	9.60 (7.97-11.20)	9.70 (8.00-11.20)	9.20 (7.60-10.95)	0.061
ALT (U/L)	20.30 (13.50-33.30)	20.00 (13.30-32.10)	22.20 (15.00-39.70)	0.001
AST (U/L)	17.30 (13.10-24.10)	17.00 (13.00-23.30)	19.00 (14.75-28.00)	0.003
Albumin (g/L)	40.80 (38.40-43.20)	40.70 (38.30-43.20)	41.10 (38.75-43.20)	0.001
Scr (μmol/L)	58.90 (49.10-70.03)	57.80 (48.70-68.20)	66.90 (54.50-86.60)	<0.001
eGFR (mL/min/1.73 m2)	104.47 ± 27.87	106.08 ± 23.27	96.05 ± 44.02	<0.001
SUA (μmol/L)	289.00 ± 91.72	264.96 ± 68.45	415.46 ± 94.91	<0.001
TG (mmol/L)	1.88 (1.30-2.75)	1.78 (1.24-2.64)	2.48 (1.73-3.96)	<0.001
TC (mmol/L)	4.92 ± 1.18	4.89 ± 1.16	5.07 ± 1.26	0.016
HDL (mmol/L)	1.12 (0.93-1.36)	1.13 (0.94-1.38)	1.03 (0.89-1.24)	<0.001
LDL (mmol/L)	2.76 (2.20-3.42)	2.77 (2.21-3.43)	2.72 (2.09-3.35)	0.329
ZJU index	42.61 ± 7.28	42.14 ± 7.00	45.09 ± 8.20	<0.001

**Table 2:** Baseline characteristics by ZJU index quantiles.

Characteristic	Q1	Q2	Q3	Q4	*P* value
Age (years)	58.07 ± 9.97	56.84 ±10.35	54.57 ± 11.42	50.35 ± 12.77	<0.001
Sex					0.831
Female	170 (38.37%)	171 (38.60%)	172 (38.83%)	182 (41.08%)	
Male	273 (61.63%)	272 (61.40%)	271 (61.17%)	261 (58.92%)	
Education					0.211
Below high school	229 (51.69%)	217 (48.98%)	231 (52.14%)	203 (45.82%)	
High school and above	214 (48.31%)	226 (51.02%)	212 (47.86%)	240 (54.18%)	
Annual household income					0.048
≤100000	267 (60.27%)	242 (54.63%)	237 (53.50%)	227 (51.24%)	
>100000	176 (39.73%)	201 (45.37%)	206 (46.50%)	216 (48.76%)	
BMI (kg/m2)	22.08 ± 2.17	24.12 ± 2.34	25.62 ± 2.70	27.78 ± 4.15	<0.001
Hypertension					0.002
No	263 (59.37%)	220 (49.66%)	223 (50.34%)	210 (47.40%)	
Yes	180 (40.63%)	223 (50.34%)	220 (49.66%)	233 (52.60%)	
Dyslipidemia					<0.001
No	341 (76.98%)	306 (69.07%)	257 (58.01%)	232 (52.37%)	
Yes	102 (23.02%)	137 (30.93%)	186 (41.99%)	211 (47.63%)	
Coronary heart disease					0.540
No	406 (91.65%)	409 (92.33%)	410 (92.55%)	417 (94.13%)	
Yes	37 (8.35%)	34 (7.67%)	33 (7.45%)	26 (5.87%)	
FPG (mmol/L)	7.51 ± 2.15	9.60 ± 2.52	11.07 ± 3.00	13.05 ± 3.27	<0.001
2hPG (mmol/L)	15.87 ± 4.74	18.55 ± 5.01	19.87 ± 5.02	21.41 ± 4.91	<0.001
HbA1c (%)	8.50 (7.00-10.50)	9.20 (7.70-10.80)	9.70 (8.25-11.25)	10.50 (9.20-11.95)	<0.001
ALT (U/L)	15.00 (10.30-22.00)	18.30 (12.60-28.00)	23.20 (15.60-39.50)	28.50 (18.85-51.35)	<0.001
AST (U/L)	16.20 (13.00-21.00)	16.10 (13.00-22.10)	18.30 (13.70-25.30)	19.00 (13.50-28.70)	<0.001
Albumin (g/L)	40.40 (37.90-42.60)	40.80 (38.20-43.40)	40.90 (38.70-43.20)	41.10 (38.85-43.35)	0.174
Scr (μmol/L)	59.90 (50.20-70.60)	59.90 (50.80-70.60)	58.90 (48.65-70.20)	56.80 (47.55-68.50)	0.081
eGFR (mL/min/1.73 m2)	101.10 ± 33.93	102.98 ± 24.93	104.91 ± 25.93	108.90 ± 25.19	<0.001
SUA (μmol/L)	272.79 ± 82.81	279.90 ± 85.57	295.39 ± 93.78	307.91 ± 99.93	<0.001
TG (mmol/L)	1.29 (0.97-1.79)	1.69 (1.26-2.39)	2.07 (1.50-2.77)	2.84 (2.01-4.79)	<0.001
TC (mmol/L)	4.57 ± 1.05	4.77 ± 1.08	4.91 ± 1.08	5.40 ± 1.32	<0.001
HDL (mmol/L)	1.22 (1.02-1.54)	1.15 (0.96-1.39)	1.10 (0.92-1.31)	1.01 (0.85-1.21)	<0.001
LDL (mmol/L)	2.58 (2.10-3.12)	2.79 (2.20-3.41)	2.81 (2.30-3.46)	2.91 (2.21-3.66)	<0.001
ZJU index	34.77 ± 2.32	39.94 ± 1.18	44.02 ± 1.15	51.72 ± 7.11	<0.001
Hyperuricemia					<0.001
No	394 (88.94%)	389 (87.81%)	363 (81.94%)	343 (77.43%)	
Yes	49 (11.06%)	54 (12.19%)	80 (18.06%)	100 (22.57%)	

### 3.2 Correlation analysis

**Table 3** indicates that the ZJU index was significantly associated with all examined variables in Spearman analyses, showing positive correlations with BMI, FPG, 2hPG, HbA1c, ALT, AST, TG, TC, LDL, eGFR, SUA, income, hypertension, dyslipidemia, and hyperuricemia, and negative correlations with age, Scr, and HDL (all P<0.05). Sex, education, and coronary heart disease showed no meaningful associations. After adjustment for age and sex, these correlations were modestly attenuated but largely preserved; however, income, Scr, eGFR, albumin, and LDL were no longer statistically significant.

**Table 3:** Correlation of the ZJU index with clinical parameters.

Variables	Non-adjusted	Adjusted for age and sex
Age (years)	-0.244**	–
Sex	-0.021	–
Education	0.030	-0.026
Annual household income	0.061*	0.006
BMI (kg/m2)	0.622**	0.559**
Hypertension	0.078**	0.167**
Dyslipidemia	0.197**	0.174**
Coronary heart disease	-0.031	0.011
FPG (mmol/L)	0.620**	0.547**
2hPG (mmol/L)	0.386**	0.339**
HbA1c (%)	0.299**	0.189**
ALT (U/L)	0.380**	0.206**
AST (U/L)	0.137**	0.068**
Albumin (g/L)	0.075**	0.025
Scr (μmol/L)	-0.076**	-0.005
eGFR (mL/min/1.73 m2)	0.197**	-0.007
SUA (μmol/L)	0.159**	0.149**
TG (mmol/L)	0.524**	0.492**
TC (mmol/L)	0.256**	0.233**
HDL (mmol/L)	-0.257**	-0.223**
LDL (mmol/L)	0.124**	0.037
yperuricemia	0.135**	0.132**

*P<0.05 **P<0.01.

### 3.3 Association between the ZJU Index and hyperuricemia

**Figure 1:**
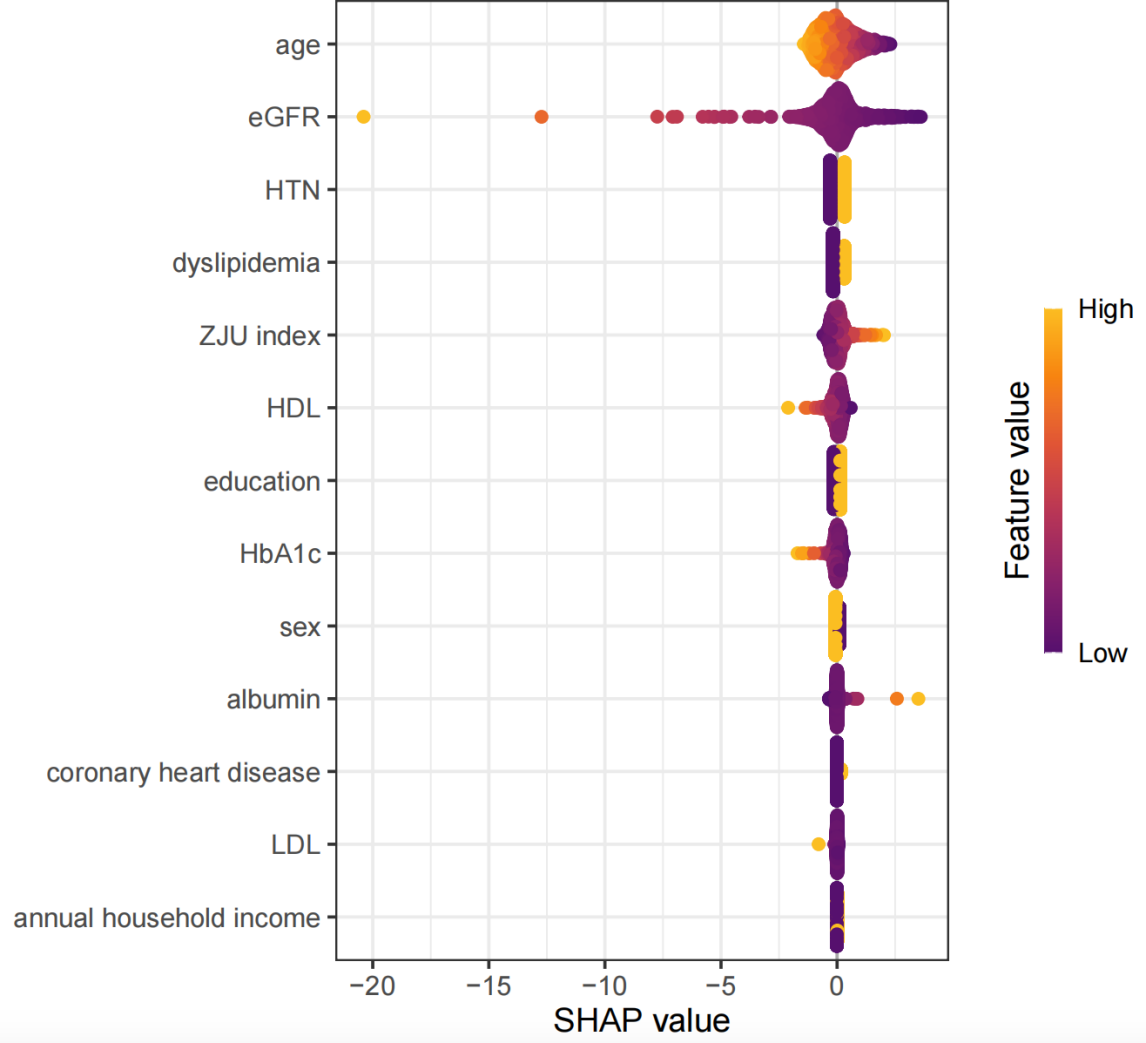
SHAP summary plot of associated features for hyperuricemia.

**Figure 2:**
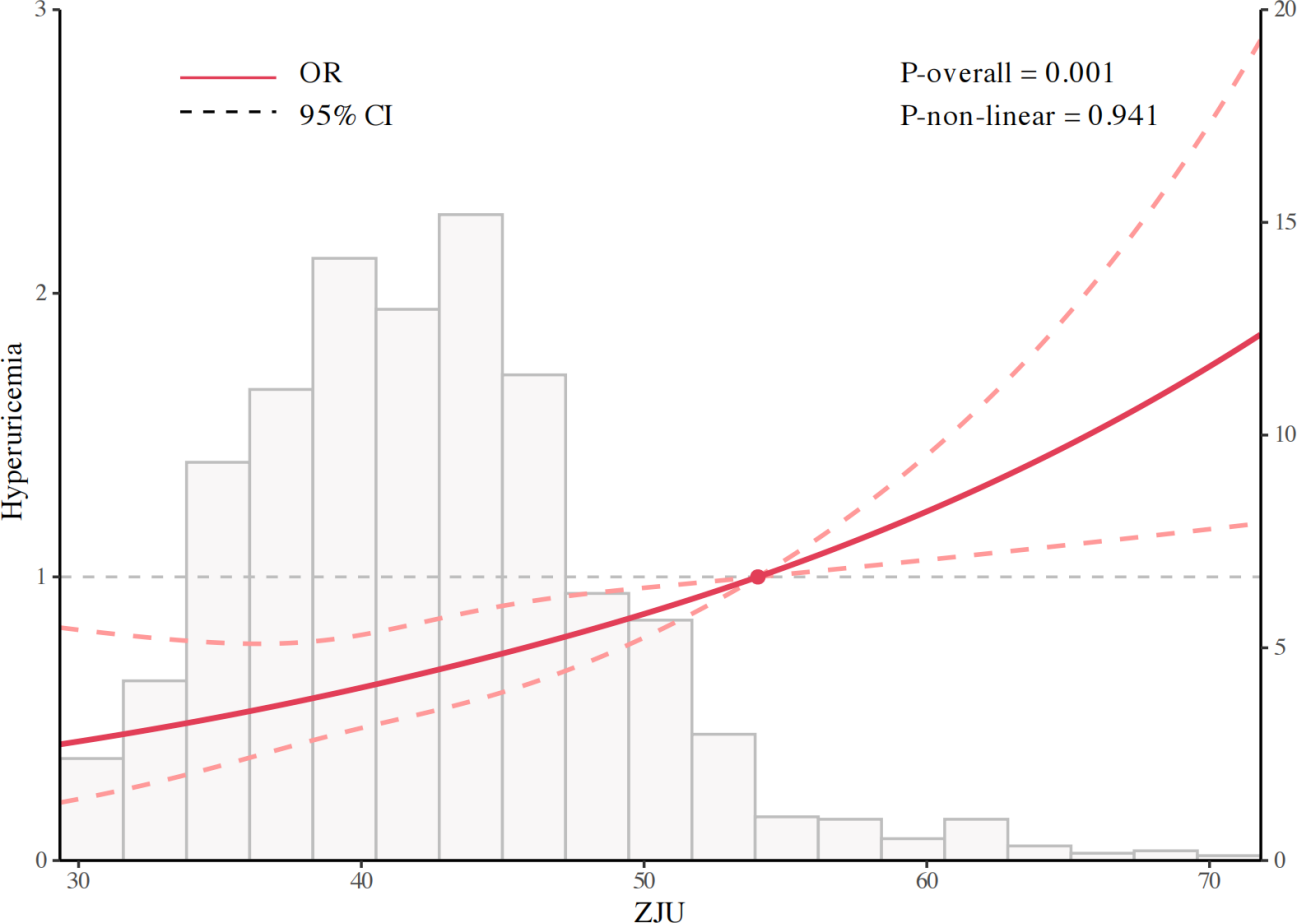
Dose-response relationship between the ZJU index and hyperuricemia.

The associations between the ZJU index and hyperuricemia were consistent across progressively adjusted models (**Table 4**). In the crude model (Model 1), the ZJU index was strongly associated with higher odds of hyperuricemia (OR = 1.050, 95%CI: 1.033-1.067, P<0.001). This association remained largely unchanged after adjustment for demographic factors in Model 2 (OR = 1.046, 95%CI: 1.028-1.063, P<0.001) and persisted in the fully adjusted model (Model 3: OR = 1.036, 95%CI: 1.017-1.056, P<0.001). Similar patterns were observed when the ZJU index was standardized, with each standard deviation (SD) increment associated with increased odds of hyperuricemia across all models (Model 1: OR = 1.423; Model 2: OR = 1.384; Model 3: OR = 1.294; all P<0.001). In quartile analyses, individuals in the highest quartile had significantly higher odds compared with the lowest quartile (Model 3: OR = 1.629, 95%CI: 1.043-2.545, P = 0.032), with evidence of a dose-response relationship (P for trend=0.008). SHAP analyses identified age and eGFR as the dominant contributor to prevalence of hyperuricemia. Additionally, the ZJU index also demonstrated a consistent association with higher hyperuricemia prevalence (**Figure 1**). Consistently, restricted cubic spline analysis indicated a significant, predominantly linear association between the ZJU index and prevalence of hyperuricemia (P overall=0.001; P non-linear=0.941) (**Figure 2**). The ZJU index was also positively associated with SUA levels across all models (**Table 5**). The effect size attenuated but remained statistically significant after full adjustment (β=2.062 in Model 1, 1.909 in Model 2, and 1.187 in Model 3; all P<0.001). Each SD increase in the ZJU index was also associated with a stepwise elevation in SUA (Model 1: β=15.017; Model 2: β=13.900; Model 3: β=8.646; all P<0.001).

**Table 4:** Logistic regression of the ZJU index and hyperuricemia.

Hyperuricemia	OR 95%CI		
	Model 1	Model 2	Model 3
ZJU index	1.050 (1.033, 1.067) <0.001	1.046 (1.028, 1.063) <0.001	1.036 (1.017, 1.056) <0.001
Each SD increase	1.423 (1.266, 1.599) <0.001	1.384 (1.225, 1.563) <0.001	1.294 (1.128, 1.484) <0.001
Quantiles			
Quartile 1	reference	reference	reference
Quartile 2	1.116 (0.740, 1.684) 0.600	1.097 (0.726, 1.656) 0.661	0.965 (0.619, 1.503) 0.874
Quartile 3	1.772 (1.208, 2.599) 0.003	1.727 (1.173, 2.542) 0.006	1.438 (0.938, 2.204) 0.095
Quartile 4	2.344 (1.617, 3.398) <0.001	2.169 (1.478, 3.184) <0.001	1.629 (1.043, 2.545) 0.032
*P* for trend	<0.001	<0.001	0.008

OR, odds ratio; 95% CI, 95% confidence interval; ZJU index, Zhejiang University index; SD, standard deviation.

Model 1: non-adjusted.

Model 2: adjusted for age, sex, education, and annual household income.

Model 3: adjusted for age, sex, education, annual household income, hypertension, dyslipidemia, coronary heart disease, HbA1c, albumin, eGFR, LDL, and HDL.

**Table 5:** Linear regression of the ZJU index and SUA levels.

SUA levels	β 95%CI		
	Model 1	Model 2	Model 3
ZJU index	2.062 (1.483, 2.641) <0.001	1.909 (1.322, 2.495) <0.001	1.187 (0.595, 1.780) <0.001
Each SD increase	15.017 (10.801, 19.232) <0.001	13.900 (9.629, 18.172) <0.001	8.646 (4.331, 12.962) <0.001
Quantiles			
Quartile 1	reference	reference	reference
Quartile 2	7.110 (-4.845, 19.064) 0.244	6.545 (-5.083, 18.174) 0.270	-0.459 (-11.591, 10.673) 0.936
Quartile 3	22.606 (10.651, 34.561) <0.001	21.466 (9.765, 33.168) <0.001	9.956 (-1.542, 21.455) 0.090
Quartile 4	35.122 (23.167, 47.076) <0.001	32.762 (20.770, 44.754) <0.001	16.911 (4.598, 29.225) 0.007
*P* for trend	<0.001	<0.001	0.002

OR, odds ratio; 95% CI, 95% confidence interval; ZJU index, Zhejiang University index; SD, standard deviation.

Model 1: non-adjusted.

Model 2: adjusted for age, sex, education, and annual household income.

Model 3: adjusted for age, sex, education, annual household income, hypertension, dyslipidemia, coronary heart disease, HbA1c, albumin, eGFR, LDL, and HDL.

In our primary analysis, we used sex-specific thresholds. Importantly, recent international guidance-such as recommendations from the American College of Rheumatology (ACR) and the European League Against Rheumatism (EULAR)-has emphasized unified urate targets rather than separate sex-based thresholds when considering clinically relevant outcomes. To address this concern, we performed sensitivity analyses using a unified SUA cutoff for both sexes (≥360 μmol/L). The association between the ZJU index and hyperuricemia prevalence remained materially unchanged, with effect estimates of similar magnitude and direction across all models (**Supplementary Material**: **Supplementary Table 1**). On the other hand, considering the potential role of estrogen in uric acid excretion, we conducted sensitivity analyses among females by stratifying participants into <50 and ≥50 years groups as a proxy for menopausal status. The results were also consistent with the primary findings (**Supplementary Material**: **Supplementary Table 2**).

### 3.4 Subgroup and ROC analysis

**Figure 3:**
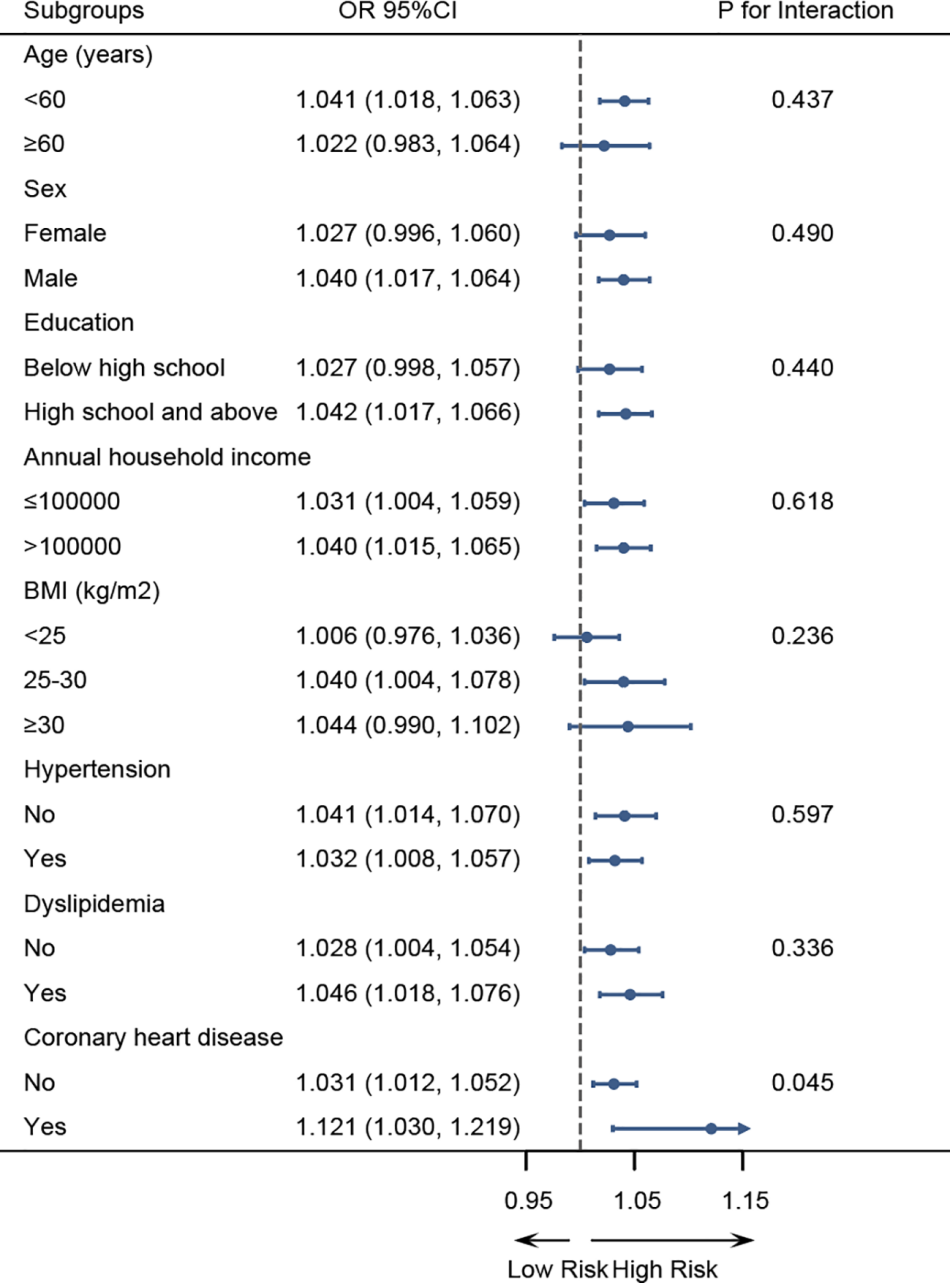
Subgroup analyses of the association between the ZJU index and hyperuricemia.

**Figure 4:**
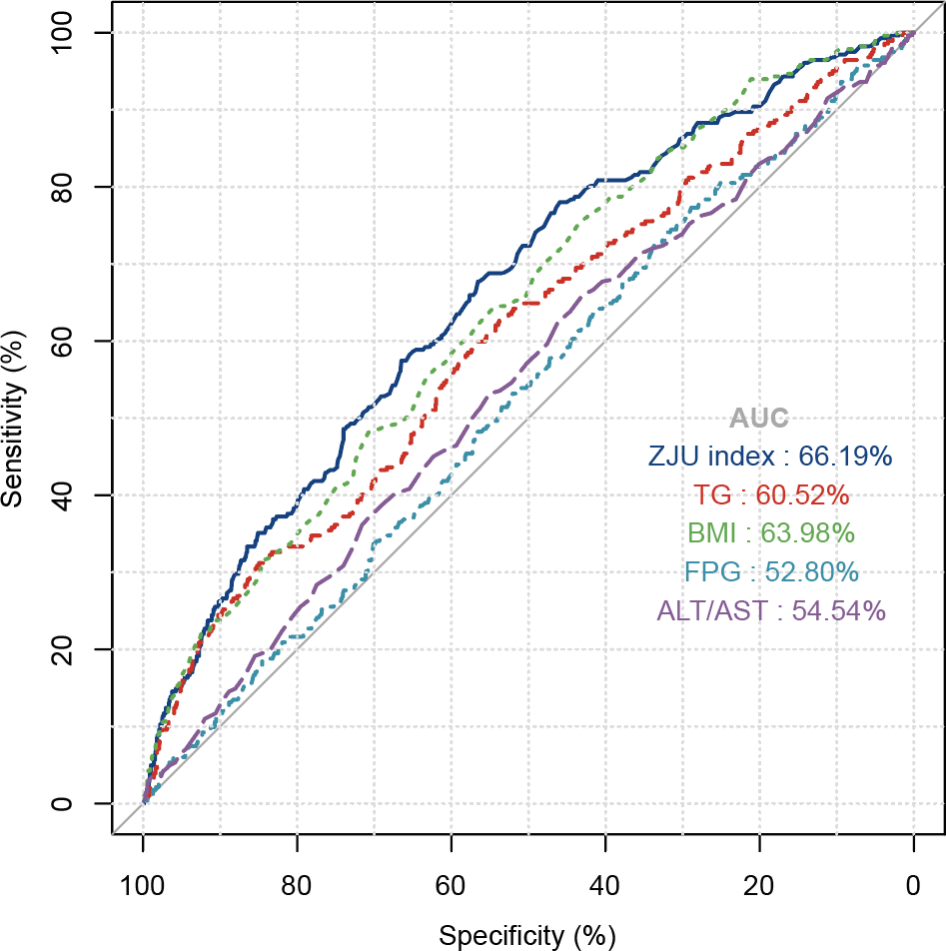
ROC curve comparison among the ZJU index and its components for hyperuricemia.

In subgroup analyses, the positive association between the ZJU index and hyperuricemia remained consistent across strata of age, sex, education level, annual household income, BMI, hypertension, and dyslipidemia, with no significant interactions observed (all P for interaction>0.05) (**Figure 3**). Notably, a significant interaction was detected for coronary heart disease (P for interaction=0.045), with a stronger association observed among T2DM participants with coronary heart disease (OR = 1.121, 95%CI: 1.030-1.219) compared with those without. We further compared the discrimination ability for hyperuricemia between the ZJU index and its individual components (BMI, FPG, TG, and ALT/AST) (**Figure 4**). ROC analyses showed ZJU index has the highest area under curve (AUC) for hyperuricemia (66.19%), compared with BMI (63.98%), FPG (52.80%), TG (60.52%), and ALT/AST (54.54%).

## 4 Discussion

Our study indicated that the ZJU index was positively and linearly associated with the prevalence of hyperuricemia in T2DM adults. Subgroup analyses further indicated that this association was more pronounced among individuals with coronary heart disease.

The ZJU index, as an integrative metabolic indicator, combines four readily accessible clinical parameters-BMI, FPG, TG, and ALT/AST ratio-to capture the composite state of insulin resistance, hepatic steatosis, and metabolic dysfunction. Previous studies have also demonstrated its strong performance in identifying fatty liver, particularly in Chinese populations (20, 21). Notably, its discriminative ability extends beyond the general population to individuals with T2DM (9, 10). In contrast to prior ZJU studies that primarily examined liver-related phenotypes or broader metabolic endpoints, this study adds incremental value by treating hyperuricemia and SUA level as primary outcomes, delineating both prevalence and dose-response patterns across the entire ZJU index range, probing effect modification by clinically relevant comorbidity (coronary heart disease), and demonstrating that the composite ZJU index discriminates hyperuricemia more effectively than any of its individual components. Mechanistically, each component of the ZJU index is closely linked to hyperuricemia. Specifically, hypertriglyceridemia is frequently accompanied by elevated free fatty acids (FFAs) and lipotoxicity, which may induce oxidative stress and inflammation, thereby contributing to increased purine turnover and uric acid production (22, 23). Hyperglycemia, by contrast, appears to influence uric acid metabolism predominantly through insulin resistance and compensatory hyperinsulinemia (24–26). Insulin has been shown to upregulate urate transporters in the renal proximal tubule, including URAT1 and GLUT9, thereby enhancing urate reabsorption and reducing its excretion (24, 27, 28). An elevated ALT/AST ratio, indicative of hepatocellular injury or steatosis, further implicates hepatic dysfunction, which is also central to uric acid synthesis and clearance (29, 30). Consistent with this, MASLD has been shown to be independently associated with an increased risk of hyperuricemia in patients with T2DM, even after adjustment for other metabolic factors (31, 32). Higher ZJU index values are likely to identify T2DM individuals with substantial hepatic fat accumulation, who are predisposed to hyperuricemia due to compounded effects of hepatic dysfunction, exacerbated insulin resistance, and systemic inflammation and oxidative stress. On the other hand, the hepato-metabolic dysregulation may also be linked to gut microbiota metabolic reprogramming (33). Gut microbiota metabolic reprogramming can promote systemic inflammation and insulin resistance via short-chain fatty acid metabolism, bile acid metabolism, and endotoxemia, thereby providing a potential mechanistic pathway (33). In subgroup analyses, we observed a statistically significant interaction between the ZJU index and coronary heart disease. First, coronary heart disease and hyperuricemia may share common pathophysiological pathways, particularly vascular endothelial dysfunction. A higher ZJU index reflects a more adverse metabolic milieu, which may further aggravate endothelial injury and promote a self-amplifying cycle between hyperuricemia and impaired vascular function (34–37). In addition, coronary heart disease is often accompanied by subclinical or overt cardiorenal impairment; altered renal hemodynamics and microvascular damage may reduce urate clearance. Second, medication use alo could contribute to the observed interaction. Patients with coronary heart disease are more likely to receive cardiovascular drugs known to influence SUA, such as diuretics, which may enhance the association in this subgroup. Reverse causality also should be considered. Mechanistically, hyperuricemia itself is known to induce hepatic steatosis and worsen insulin resistance (components of the ZJU index).

Overall, ZJU index relies solely on routinely measured clinical variables, requiring no additional cost or specialized testing, which makes it highly suitable for use in primary care. Rather than serving as a screening or diagnostic substitute for hyperuricemia, the value of the ZJU index in this context lies in its ability to capture the integrated hepato-metabolic disturbances underlying hyperuricemia in T2DM. As an established composite marker, it may help characterize the interplay between hepatic dysfunction, insulin resistance, and lipid metabolism that contributes to elevated uric acid levels.

Several limitations should be considered. First, because of the single-center cross-sectional/retrospective design, causality cannot be inferred, and the observed associations reflect correlations rather than cause-effect relationships. Patient recruitment in a clinical setting may introduce selection/referral bias (including Berkson’s paradox). The prevalence of hyperuricemia and metabolic profiles may also be influenced by local dietary habits and genetic background, so findings may not be fully generalizable to Western or multi-ethnic populations. Second, residual confounding cannot be excluded. Although hyperuricemia is defined to include current urate-lowering medication use, several T2DM-associated drug classes (e.g., SGLT2 inhibitors, diuretics, and lipid-lowering agents) may still affect SUA; Moreover, urate-lowering therapy can reduce SUA concentrations and alter SUA distributions, potentially causing residual misclassification. In addition, we did not collect detailed dietary and lifestyle data, particularly information on purine-rich food intake, fructose consumption, alcohol intake, and physical activity. These factors are known to profoundly influence SUA levels and may introduce dietary and lifestyle-related confounding. Third, the ZJU index integrates multiple clinical indicators; however, the impact of short-term temporal variability on the association with hyperuricemia remains unknown.

## 5 Conclusion

In conclusion, this retrospective cross-sectional study identified an independent association between the ZJU index and the prevalence of hyperuricemia among adults with T2DM. Given the cross-sectional design, temporal relationships cannot be determined, and therefore, no causal or predictive inferences should be drawn.

## Data Availability

The corresponding author will make available the datasets generated and/or analyzed in this study upon reasonable request.
