# Supplementary material for: Association between the Zhejiang University index and hyperuricemia among adults with type 2 diabetes in China
Source: Front Endocrinol (Lausanne). 2026 Apr 23;17:1836849. doi: 10.3389/fendo.2026.1836849 (PMC13149066; doi:10.3389/fendo.2026.1836849)
Supplement: Supplementary file 1 [file Supplementaryfile1.docx]

**Supplementary Material**

| **Table S1** Sensitivity analyses using a unified SUA cutoff (≥360 μmol/L for both sexes). | | | |
| --- | --- | --- | --- |
| Hyperuricemia | OR 95%CI | | |
|  | Model 1 | Model 2 | Model 3 |
| ZJU index | 1.046 (1.030, 1.062) <0.001 | 1.042 (1.025, 1.059) <0.001 | 1.034 (1.015, 1.053) <0.001 |
| Each SD increase | 1.389 (1.243, 1.553) <0.001 | 1.350 (1.201, 1.518) <0.001 | 1.275 (1.118, 1.454) <0.001 |
| Quantiles |  |  |  |
| Quartile 1 | reference | reference | reference |
| Quartile 2 | 1.113 (0.769, 1.611) 0.571 | 1.096 (0.755, 1.592) 0.630 | 0.997 (0.669, 1.487) 0.990 |
| Quartile 3 | 1.669 (1.176, 2.367) 0.004 | 1.616 (1.132, 2.305) 0.008 | 1.365 (0.924, 2.018) 0.118 |
| Quartile 4 | 2.115 (1.504, 2.974) <0.001 | 1.977 (1.384, 2.823) <0.001 | 1.557 (1.031, 2.351) 0.035 |
| ***P*** for trend | <0.001 | <0.001 | 0.012 |
| OR: odds ratio; 95% CI: 95% confidence interval; ZJU index: Zhejiang University index; SD: standard deviation  Model 1: non-adjusted.  Model 2: adjusted for age, sex, education, and annual household income.  Model 3: adjusted for age, sex, education, annual household income, hypertension, dyslipidemia, coronary heart disease, HbA1c, albumin, eGFR, LDL, and HDL. | | | |

| **Table S2** Sensitivity analyses in women stratified by <50 vs. ≥50 years as a proxy for menopausal status. | | |
| --- | --- | --- |
| Hyperuricemia | OR 95%CI P value | |
|  | Age <50 years | Age ≥50 years |
| ZJU index | 1.063 (1.006, 1.124) 0.031 | 1.027 (0.993, 1.061) 0.121 |
| Each SD increase | 1.523 (1.039, 2.231) 0.031 | 1.198 (0.954, 1.504) 0.121 |
| Quantiles |  |  |
| Quartile 1 | reference | reference |
| Quartile 2 | 2.190 (0.363, 13.219) 0.393 | 1.144 (0.569, 2.298) 0.706 |
| Quartile 3 | 0.348 (0.030, 4.074) 0.401 | 1.526 (0.778, 2.996) 0.219 |
| Quartile 4 | 5.552 (1.144, 26.939) 0.033 | 1.567 (0.792, 3.099) 0.197 |
| ***P*** for trend | 0.021 | 0.134 |
| OR: odds ratio; 95% CI: 95% confidence interval; ZJU index: Zhejiang University index; SD: standard deviation  Adjusted for age, education, annual household income, hypertension, dyslipidemia, coronary heart disease, HbA1c, albumin, eGFR, LDL, and HDL. | | |
